# Role of novel biomarkers urinary NGAL and MCP-1 in predicting progression of diabetic kidney disease in type 2 DM

**DOI:** 10.1080/0886022X.2025.2563671

**Published:** 2025-09-30

**Authors:** Shilna Muttickal Swaminathan, Mohan Varadanayakanahalli Bhojaraja, Indu Ramachandra Rao, Attur Ravindra Prabhu, Shivashankara Kaniyoor Nagri, Dharshan Rangaswamy, Srinivas Vinayak Shenoy, Sahana Shetty, Ravindra Maradi, Ankur Gupta, Kirthinath Ballala, Shankar Prasad Nagaraju

**Affiliations:** ^a^ Department of Nephrology, Kasturba Medical College, Manipal, Manipal Academy of Higher Education, Manipal, Karnataka, India; ^b^ Department of Medicine, Kasturba Medical College, Manipal, Manipal Academy of Higher Education, Manipal, Karnataka, India; ^c^ Department of Endocrinology, Kasturba Medical College, Manipal, Manipal Academy of Higher Education, Manipal, Karnataka, India; ^d^ Department of Biochemistry, Kasturba Medical College, Manipal, Manipal Academy of Higher Education, Manipal, Karnataka, India; ^e^ Department of Nephrology, Palmerston North Hospital, New Zealand; ^f^ Department of Community Medicine, Kasturba Medical College, Manipal, Manipal Academy of Higher Education, Manipal, Karnataka, India

**Keywords:** NGAL, MCP-1, diabetic kidney disease, proteinuria

## Abstract

The prediction of rapid progression in diabetic kidney disease (DKD) remains a global challenge. This study evaluated the prognostic value of two noninvasive biomarkers neutrophil gelatinase-associated lipocalin (NGAL) and monocyte chemoattractant protein-1 (MCP-1) in identifying rapid DKD progression. In this prospective observational study, 145 T2DM patients with DKD (October 2021–June 2024) were categorized as rapid or nonrapid progressors based on an eGFR decline >5 mL/1.73 m^2^/year. Baseline urinary(u) NGAL and MCP-1 were measured by ELISA. Clinical profiles, risk factors, and predictive utility of the biomarkers were analyzed. During a median follow-up of 1.3 years, 38.6% were rapid progressors. Hypertension, cardiovascular disease, elevated SBP, high fasting blood sugar, and higher urinary albumin-to-creatinine ratio (uACR) were significantly associated with rapid progression (*p* < 0.05). Median uNGAL and uMCP-1 levels were higher in rapid progressors (57.6 vs 28.2 ng/ml; 469 vs 220 pg/ml; *p* = 0.01) and increased with albuminuria severity (*p* = 0.03 and *p* = 0.01). Multivariate analysis identified uNGAL, uMCP-1, and uACR as independent risk factors. uMCP-1 showed the highest diagnostic accuracy (AUC 0.94; 94.1% sensitivity; 89.2% specificity at 381.2 pg/ml). uNGAL had an AUC of 0.86 (83.4% sensitivity; 77.3% specificity at 39.8 ng/ml). A combined panel of uMCP-1, uNGAL, and uACR further improved predictability (AUC 0.96). Patients with elevated uNGAL and uMCP-1 levels experienced a greater incidence of rapid progression similar to uACR. uMCP-1 exhibited better diagnostic accuracy than did uNGAL and uACR, with better sensitivity and specificity in identifying rapid progressors. The combination of three noninvasive biomarkers had further improved predictability.

## Introduction

Diabetic kidney disease (DKD) is the most common microvascular complication that occurs in 20%-40% of patients with type 2 diabetes mellitus and contributes to cardiovascular morbidity and mortality, which reduce health-related quality of life [1]. Renal function in patients with DKD gradually declines over time; however, a significant proportion of patients progress rapidly within months, leading to end-stage kidney disease (ESKD) requiring renal replacement therapies [2,3]. Clinical factors associated with the rapid decline in renal function have not been extensively studied, although proteinuria is considered to be a significant contributing factor.

DKD is a progressive disease, and its pathophysiology is complex, affecting almost all nephron structures. Early diagnosis plays a crucial role in preventing the progression of DKD. However, owing to the heterogeneity of its clinical presentation and multifactorial pathogenesis, early prediction of rapid progression remains a clinical challenge globally [4]. Albuminuria and the eGFR are the gold standard biomarkers of DKD in routine clinical practice [5]. Nevertheless, the increasing prevalence of nonproteinuric progression of DKD and changes in serum creatinine are delayed until significant loss of renal function, and the sensitivity and specificity of existing biomarkers are limited in predicting disease progression [5]. These findings suggest that tubulointerstitial involvement may precede glomerular injury. More recently, the role of chronic inflammation has also been recognized in DKD pathogenesis. Recognizing these constraints and exploring potential biomarkers are essential for advancing clinical applications that can aim to increase diagnostic accuracy and predictive capabilities.

The study of biomarkers in DKD is still in a growing stage, and information about the timely diagnosis of DKD is incomplete. Recent evidence exploring novel biomarkers of renal tubulointerstitial injury and inflammation is vital for achieving more precise predictions of the progression of DKD. Among the broadly classified novel biomarkers, neutrophil gelatinase-associated lipocalin (NGAL) [6] and monocyte chemoattractant protein-1 (MCP-1) are promising novel biomarkers for predicting progression [7]. NGAL signifies tubular injury, whereas MCP-1 indicates inflammatory changes.

NGAL is a member of the lipocalin superfamily and is structurally characterized by sequences of 178 amino acid glycoproteins and a molecular weight of 25 kDa [8]. Although NGAL was initially identified in neutrophils, it is also present in various human tissues, such as the kidney, heart, liver, and lung. Specifically, within the kidney, it is expressed in tubular epithelial cells, indicating tubular functional capacity, and thereby, its level increases with tubular damage. Moreover, NGAL serves as a circulating biomarker intricately linked with hyperglycemia, insulin resistance, and obesity, which are all significant risk factors contributing to kidney injury associated with DKD [9].

More recently, inflammatory signaling pathways have been recognized to play a significant role in the progression of DKD. MCP-1 (CCL2), a potent monocyte attractant belonging to the CC subfamily of chemokines, is produced by mononuclear leukocytes, epithelial cells of cortical tubules, and podocytes [6,7]. MCP-1 has been shown to facilitate macrophage accumulation and mediate interstitial inflammation and consequent fibrosis in DKD [10]. Both animal and human studies have examined the levels of circulating and urinary MCP-1 in DKD patients and reported that these levels are significantly correlated with DKD pathogenesis [11].

However, studies on the diagnostic accuracy of these biomarkers with respect to disease severity are scarce. This is the first study in the Indian population to assess the diagnostic role of a combination of two key pathogenic pathway indicators, NGAL and MCP-1, in predicting rapid DKD progression.

The present study aimed to evaluate the diagnostic utility of two novel noninvasive biomarkers, urinary NGAL and MCP-1, in differentiating between rapid progressors and nonrapid progressors. We also explored the prevalence, clinical profile, and potential risk factors associated with rapid progression.

## Materials and methods

### Study population

This was a prospective observational study conducted at Kasturba Medical College, Manipal, MAHE, Manipal, India. A total cohort of 145 T2DM patients with DKD from October 2021 to June 2024 was studied after approval from the institutional ethics committee (IEC-421-2021) and Clinical Trails Registry India (CTRI/2021/09/036450). Informed consent was obtained from all participants.

### Inclusion criteria

All T2DM patients aged above 18 years who were clinically diagnosed with DKD as per the KDIGO guidelines (CKD stages 1–3) were included in this study.

### Exclusion criteria

DKD patients with superimposed nondiabetic renal disease, advanced CKD stages (CKD 4 and CKD 5), autoimmune disease, active urinary tract infection, renal calcular disease, cancer, and less than 6 months of follow-up were excluded.

### Data collection

Baseline clinical and demographic data, including age; height; weight; body mass index; sex; waist–hip ratio; abdominal circumference; and biochemical laboratory parameters, such as the urine albumin (ALB) creatinine level and the serum levels of blood sugar, creatinine, urea, total cholesterol, triglycerides, bicarbonate, and HbA1c, were recorded for all patients according to the protocol. Blood pressure was measured at the hospital *via* a manual mercury sphygmomanometer. HbA1c was analyzed *via* high-performance liquid chromatography on a Bio-Rad D100 system. Total cholesterol (colorimetric method), triglycerides (enzymatic method), urea (kinetic test with urease and glutamate dehydrogenase), creatinine (Jaffe method), and bicarbonate (CO2-L assay) were measured on a Cobas 8000 analyzer (Module 701) from Roche Diagnostics. The level of the uACR was assessed *via* the immunoturbidometric method on Module 501 of the Cobas 8000 system. All the above-mentioned assays were performed according to the manufacturer’s instructions.

Following baseline assessments, patients were prospectively followed up until the end of the observation period. The outcome studied was the rapid decline in the eGFR. At the end of the follow-up, patients were categorized into rapid progressors and nonrapid progressors on the basis of a decrease in the eGFR.

### Urinary NGAL and MCP-1 assay

A single time-point estimation of urinary NGAL and MCP-1 was performed in the baseline sample. Eight milliliters of a urine sample for biomarker analysis was collected once at the time of enrollment. The urine samples were centrifuged at 3000 rpm for 10 min, and the supernatant was stored at 800 °C until analysis. A sandwich enzyme-linked immunosorbent assay (ELISA) was employed to measure the biomarkers quantitatively, including in duplicate samples. A fine test ELISA kit (Cat. No EH0012) was used for urine NGAL (uNGAL). A Diaclone ELISA kit (Cat. No 873.030.096) was used for urine MCP-1 (uMCP-1) analysis. The optical density was read by using a Thermo Scientific Multiskan FC ELISA reader. uNGAL levels are expressed in ng/ml, whereas uMCP-1 levels are expressed in pg/ml.

### Definitions

**DKD:** As per the KDIGO guidelines, DKD is defined as persistently elevated proteinuria, reduced eGFR, or both, with evidence of diabetic retinopathy/biopsy-proven diabetic nephropathy [12].

**eGFR & CKD staging:** The “CKD-EPI equation was used to estimate eGFR using the serum creatinine, GFR = 141*min (Scr/κ, 1) α * max (Scr/κ,1)-1.209 * 0.993Age *1.018(if female) ×1.159 (if black), where Scr is the serum creatinine in mg/dl, κ is 0.7 for females and 0.9 for males, α is −0.329 for females and − 0.411 for males, min indicates the minimum of Scr/κ or 1, and max indicates the maximum of Scr/κ or 1”. The patients were categorized into CKD stages according to the KDIGO criteria [13,14].

**Rapid Progressors:** Sustained decline in eGFR of > 5 mL/min per 1.73 m^2^/year using the 2009 CKD-EPI creatinine equation as per the KDIGO 2012 criteria [12].

**Proteinuria:** Based on the urine albumin–creatinine ratio (uACR), proteinuria was classified as normal to mildly increased(A1),moderately increased,(A2) or severely increased albuminuria (A3) in align with KDIGO guidelines

### Statistical analysis

The Shapiro test was used to assess the normal distribution of variables. The means ± SDs represent continuous variables, whereas the medians with interquartile ranges are used for noncontinuous variables. The percentages of categorical variables were compared *via* the chi-square test or Fisher’s exact test. We used t-tests and the Mann–Whitney U-test to compare the means and medians for continuous variables. Kruskal–Wallis ANOVA with Bonferroni analysis was used to compare the median levels of biomarkers across the various stages of proteinuria and CKD. Spearman’s correlation analysis was performed to correlate NGAL and MCP-1 with other laboratory parameters. Univariate and multivariate regression analyses were used to assess the risk factors contributing to rapid progression. Receiver operating characteristic (ROC) curves were generated, and the area under the curve (AUC) was calculated to assess the sensitivity and specificity of uNGAL and uMCP-1 in predicting the rapid progression of DKD. Post hoc power analysis was performed for one-way ANOVA and regression analysis, and the eta-square effect size was calculated and converted to Cohen’s f for power calculation for one-way ANOVA. The effect size was estimated *via* the R-squared (R^2^) values from the model for regression analysis, and *post hoc* power analysis was performed *via* an F test. A *p* value of <0.05 was considered significant. The data were analyzed *via* SPSS version 20.0.

## Results

**Figure 1.**
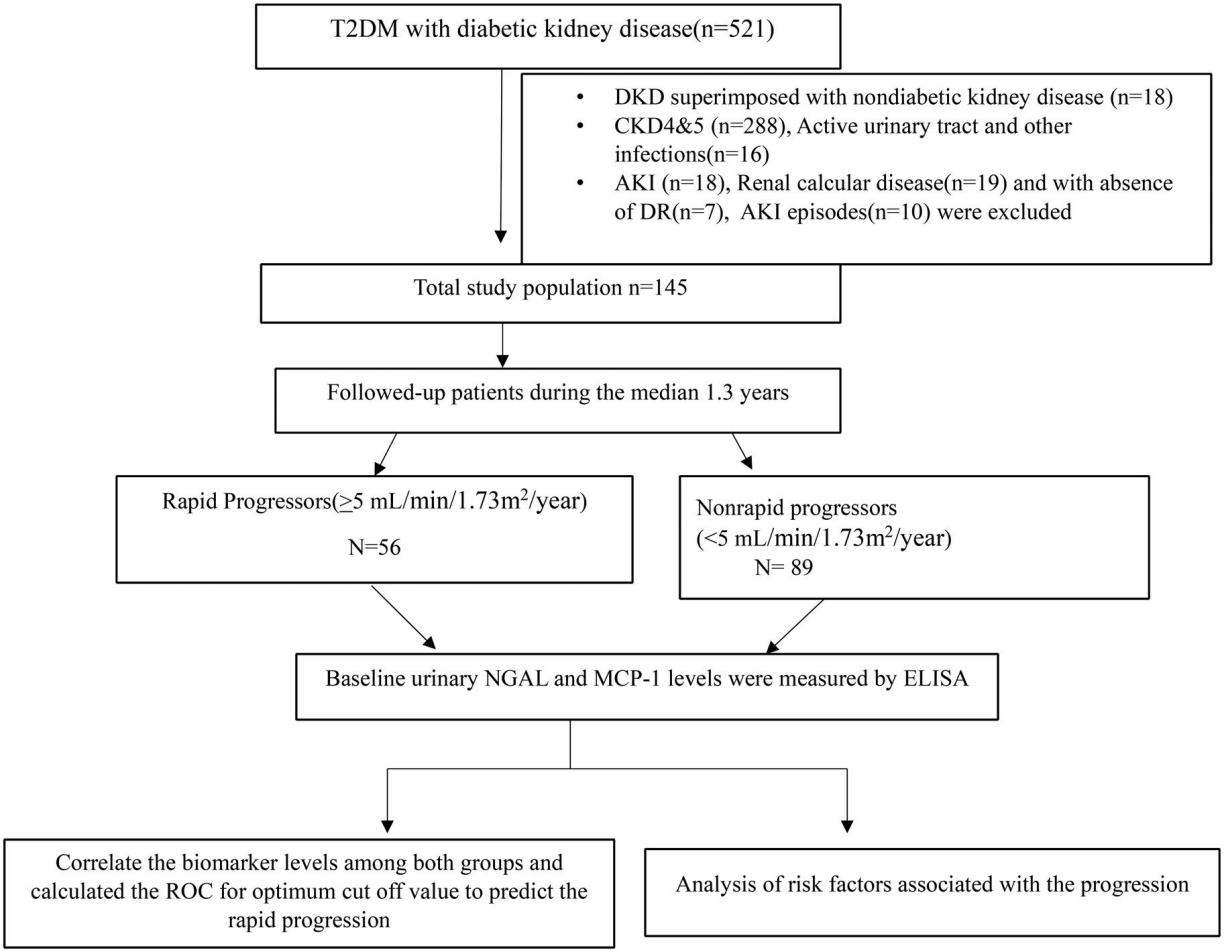
Flow diagram of the study population.

A total of 145 type 2 diabetes mellitus patients with DKD were studied, as shown in Figure 1. In our study cohort, 56 (38.6%) patients were rapid progressors, and 89 (61.3%) patients were nonrapid progressors during the median follow-up period of 1.3 years (IQR 1.0–1.6), with median decreases in eGFRs of −18.1 and −8.5 mL/min/1.73m^2^/year, respectively, and −3.3 and 0.0 mL/min/1.73 m^2^/year, respectively.

The baseline clinical characteristics of the groups are compared in Table 1. The incidences of hypertension (96.4% vs 77.5%, *p* = 0.01) and cardiovascular disease (57.1% vs 32.6%, *p* = 0.01) were significantly greater in rapid progressors than in nonprogressors. The systolic blood pressure was significantly high in rapid progressors (164 mm Hg (156–168.5) vs 140 mmHg (130–140), *p* = 0.03). Among patients with CVD, the majority were diagnosed with ischemic heart disease (81.2%), followed by LV dysfunction (8.2%) and heart failure with preserved ejection fraction (6.2%). All patients were diagnosed with diabetic retinopathy; however, the proportions of NPDR and PDR did not significantly differ among the groups, with NPDR 85.9% vs 84.4% (*p* = 0.37) and PDR 9.8% vs 6.7% (*p* = 0.64). Other clinical characteristics, including age, BMI, waist–hip ratio, abdominal circumference, smoking status, duration of diabetes/hypertension, dyslipidemia, PVD, and CVA, were not significantly different among the groups (*p* > 0.05).

**Table 1.** Baseline demographic, clinical, and laboratory characteristics.

Characteristics	Overall (*n* = 145)	Rapid progressors (*n* = 56)	Nonprogressors (*n* = 89)	*P* value
Age (years)	62 (53–67)	66 (52.5–70)	62 (53–67)	0.15
Males*, n* (%)	113 (77.9)	45 (80.5)	68 (76.4)	0.09
BMI (kg/m^2^)	24.5 (21–29.4)	25.8 (53.5–67.5)	21.8 (19.8–31.0)	0.24
Waist to Hip ratio	0.97 (0.90–1.01)	0.99 (0.89–1.01)	0.97 (0.9–1.0)	0.36
Abdominal Circumference (cm)	98 (90–107)	100 (90–108)	96.0 (90–102.8)	0.38
Smoking, *n* (%)	7 (4.9)	5 (5.6)	2 (3.6)	0.23
***Comorbidities***				
Hypertension, *n* (%)	123 (84.8)	54 (96.4)	69 (77.5)	**0.01** *
Systolic BP (mmHg)	150 (130–178)	164 (156–168.5)	140 (130–140)	**0.02** *
Diastolic BP (mmHg)	80 (75–90)	80.4 (80–90)	79.0 (70–90)	0.92
Duration of diabetes (in years)	10 (5–15)	10.2 (5–15.0)	10.0 (4.2–15.0)	0.25
Duration of Hypertension (in years)	12 (7–20)	10 (6.7–17.0)	12.0 (6.7–20.2)	0.47
Dyslipidemia, *n* (%)	86 (59.6)	34 (60.7)	52 (58.4)	0.24
NPDR, *n* (%)	123 (84.8)	48 (85.9)	75 (84.4)	0.37
PDR, *n* (%)	12 (8.2)	6 (9.8)	6 (6.7)	0.64
CVA, *n* (%)	14 (9.7)	7 (12.5)	7 (7.8)	0.46
PVD, *n* (%)	15 (15.2)	7 (12.2)	7 (9.0)	0.08
CVD, *n* (%)	61 (42.2)	32 (57.1)	29 (32.6)	**0.01** *
***Laboratory Parameters***				
HbA1C (%)	8.5 (7.2–9.8)	7.6 (6.5–9.6)	7.0 (6.0–8.9)	0.94
Fasting Blood Sugar (mg/dl)	148 (118–199)	174 (119–223)	127 (114–154)	**0.03** *
Total Cholesterol (mg/dl)	167 (130–204)	170.8 (129–210)	169 (131–199)	0.78
Triglycerides (mg/dl)	154 (114–240)	181 (125–253)	149 (108–202)	0.41
Serum creatinine (mg/dl)	1.37 (1.301–1.71)	1.43 (1.0–1.76)	1.2 (0.9–1.6)	0.78
Urea (mg/dl)	33 (25–44)	33 (26–44)	32 (24–43)	0.27
Bicarbonate (mmol/L)	21.4 (19.8–23.9)	23 (19.4–23.9)	21.3 (19–24.0)	0.48
UACR (mg/g)	440 (204–2109)	1027 (231–3608)	264 (163–674)	**0.01** *
eGFR decline (mL/min/1.73 m^2^/year)	−3.8 (−9.3, −0.8)	−12 (−18.1, −8.5)	−1.4 (−3.3,0.0)	**0.01** *
***CKD Stages***				0.65
G1	23 (15.8)	6 (11.5)	17 (19.3)	
G2	39 (26.8)	14 (25.7)	25 (28.7)	
G3a	44 (30.3)	18 (33.4)	26 (29.5)	
G3b	39 (26.8)	18 (32.1)	21 (23.6)	
**Albuminuria Stages**				**0.04** *
normal to mildly increased (A1)(<30 mg/g)	18 (12.4)	4 (7.1)	14 (15.7)	
Moderately increased (A2) (30–300 mg/g)	40 (42.7)	16 (28.7)	24 (26.3)	
Severely increased albuminuria (A3) (>300 mg/g)	87 (44.8)	36 (64.9)	51 (55.8)	

BMI, body mass index; HbA1c, glycated hemoglobin; NPDR, nonproliferative diabetic retinopathy; PDR, proliferative diabetic retinopathy; CVD, cardiovascular disease; CVA, cerebrovascular accident; PVD, peripheral vascular disease; eGFR, estimated glomerular filtration rate.

* statistically significant.

Among the biochemical parameters, only the fasting blood sugar level (174 mg/dl (119–223) vs 127 mg/dl (114–154), *p* = 0.03) and the urine albumin–creatinine ratio (1027 mg/g (231–3608) vs 264 mg/g (163–674), *p* = 0.01) were significantly higher in rapid progressors than in nonprogressors. Rapid progressors were found in 7.1% of patients with normal to mildly increased (A1), 32.1% with moderately increased, (A2), and 67.9% with severely increased albuminuria(A3), which was statistically significant (*p* = 0.04). Furthermore, the differences in the baseline serum creatinine levels and CKD stages were not statistically significant between the groups (*p* = 0.78 and *p* = 0.65, respectively); however, the majority of rapid progressors were at baseline CKD stage 3a (33.4%).

We further analyzed the baseline medication of the study population. However, there was no statistically significant difference between the groups in terms of the use of antihypertensive drugs. Calcium channel blockers remained the most commonly used treatment (47.2%), followed by renin-angiotensin-aldosterone system (RAAS) blockers (35.8%). Insulin with oral antihyperglycemic agents was prescribed in 51.7% of nonrapid progressors, 26.9% of whom were in sodium–glucose cotransporter-2 inhibitor (SGLT2i) classes.

Among the 145 patients, 66 patients (45.2%) were on SGLT2i alone or in combination with angiotensin II receptor blocker (ARB) (SGLT2i + ARB)/or ARB alone, whereas the remaining 79 (54.1%) did not receive either SGLT2 inhibitors or ARB therapy. Compared with not receiving these treatments, rapid progression was not significantly associated with treatment with SGLT2 inhibitors alone or in combination with ARBs(SGLT2i + ARB)/or ARBs alone (*p* = 0.46) (Table 2).

**Table 2.** Pattern of treatment among the study population.

Treatment	Overall	Rapid progressors	Nonprogressors	*P* value
SGLT2i alone/combination with ARB (SGLT2i + ARB)/or ARB alone, n (%)	66 (45.2)	26 (46.5)	40 (44.9)	0.79
Not on SGLT2i alone/combination with ARB (SGLT2i + ARB)/or ARB alone, n (%)	79 (54.1)	30 (53.6)	49 (55.1)	0.46

SGLT2i, sodium–glucose cotransporter-2 inhibitor; ARB, angiotensin II receptor blocker.

**Figure 2.**
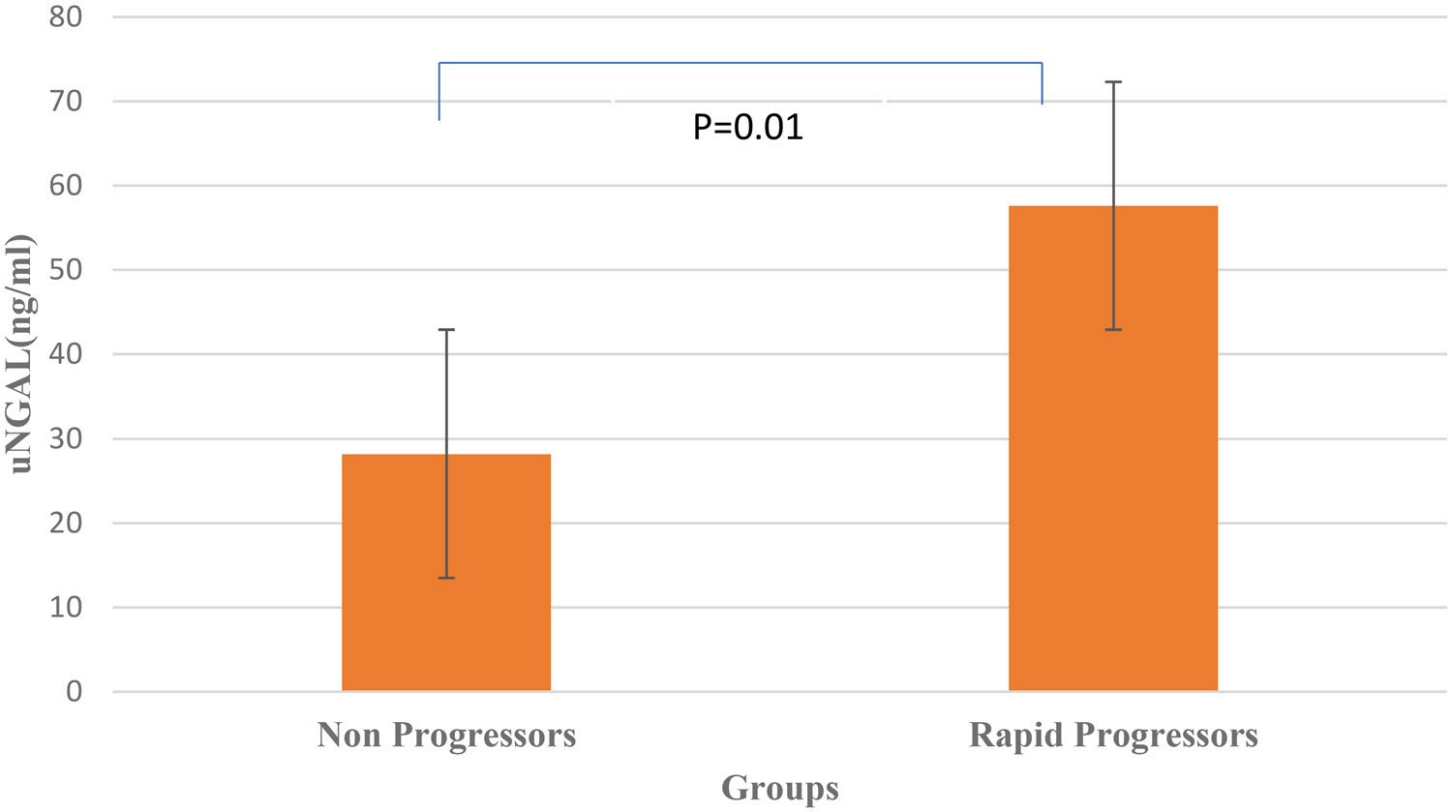
uNGAL in Rapid progressors and nonprogressors.

**Figure 3.**
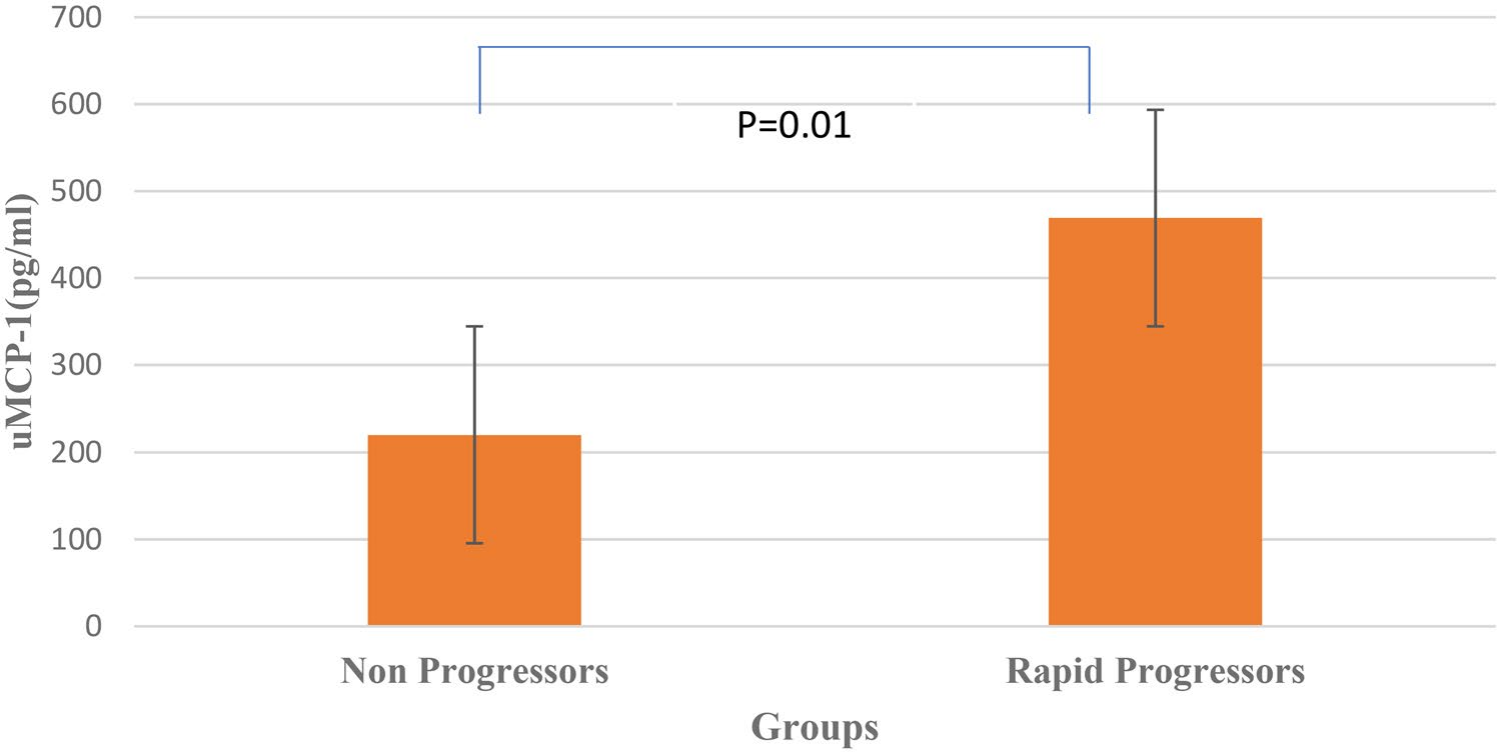
uMCP-1 in Rapid progressors and nonprogressors.

The median uNGAL was significantly higher in rapid progressors than in nonprogressors (57.6 ng/ml, IQR 41.9–73.8 vs 28.2, IQR 14.9–38.0, *p* = 0.01, respectively). uMCP-1 levels were also showed significant rise in rapid progressors than nonprogressors (469 pg/ml, IQR 405–534 vs 220, IQR 117–300, *p* = 0.01, respectively) (Figures 2 and 3). We further evaluated the levels of biomarkers at three different stages of proteinuria, as shown in Table 3. Both the uNGAL and uMCP-1 levels were found to increase gradually from A1 to A3 albuminuria group.

**Table 3.** Comparison of median uNGAL and uMCP-1 levels among different stages of proteinuria.

Biomarker	A1 (*n* = 18)	A2 (*n* = 40)	A3 (*n* = 87)	*P* value
uNGAL, ng/ml	12.6 (IQR4.7–15.6)	29.1 (IQR18.5–40)	52.3 (IQR38.3–66.5)	**0.03**
uMCP-1,pg/ml	46.5 (IQR14.4–86.7)	238 (IQR148.2, 410.4)	398 (IQR279.6–488.3)	**0.01**

IQR, Interquartile range; A1- normal to mildly increased albuminuria, A2- moderately increased albuminuria, A3- severely increased albuminuria.

Furthermore, among those with A1 albuminuria, those who exhibited rapid progression had significantly higher uNGAL and uMCP-1 levels, with median uNGAL levels of 19.3 vs 10.4 ng/ml (*p* = 0.04) and 370 vs 42.5 pg/ml (*p* = 0.01), respectively.

In the comparison of the uNGAL and uMCP-1 levels among the different stages of CKD, both were elevated as the eGFR decreased, and a significant difference was observed only between stages G1 and G3b (*p* = 0.02 and 0.01, respectively) (Table 4). The *post hoc* power analysis, which was based on the eta-square effect size for NGAL and MCP-1, indicated powers of 0.90 and 1.00, respectively, suggesting that the study had sufficient power to detect differences in biomarker levels among these groups.

**Table 4.** Comparison of uNGAL and uMCP-1 among different stages of CKD.

Biomarkers	G1 (*n* = 23)	G2 (*n* = 39)	G3a (*n* = 44)	G3b (*n* = 39)
uNGAL, ng/ml	29.6 (IQR 21.8–44.2)	31.4 (IQR19–47.7)	34 (IQR16–61.3)	48.5 (IQR 36–57.9)
uMCP-1, pg/ml	238 (IQR 167–394)	279 (IQR159–338)	289 (IQR137.3–358)	402 (IQR 298–488)
IQR, Interquartile range;G1, CKD Stage 1; G2, CKD Stage 2; G3a, CKD Stage 3a; G3b, CKD Stage 3b				

Spearman’s rank correlation coefficient was calculated for uNGAL and uMCP-1 with other biochemical parameters, as shown in Table 5. uNGAL and uMCP-1 were strongly positively correlated (*p* = 0.001) with the uACR. Spearman’s coefficient (rho) was 0.56, and for uMCP-1, it was 0.46. Similarly, a strong negative correlation was found with a decrease in the eGFR, with a rho value of −0.4 (*p* = 0.001).

**Table 5.** Spearman’s correlation analysis of uNGAL and uMCP-1.

Parameters		FBS	PPBS	HbA1C	Creatinine	Urea	uACR	eGFR decline
uNGAL	Spearman’s ρ	−0.18	0.07	0.02	**0.26**	0.17	**0.56**	**−0.46**
	p value	0.73	0.17	0.47	**0.01**	0.19	**0.01**	**0.01**
uMCP-1	Spearman’s ρ	−0.21	0.06	−0.09	**0.23**	0.04	**0.48**	**−0.4**
	p value	0.28	0.12	0.09	**0.01**	0.32	**0.01**	**0.01**

FBS, fasting blood sugar; PPBS, postprandial blood sugar; uACR, urine albumin creatinine ratio; eGFR, estimated glomerular filtration rate.

### Risk factors associated with rapid progression

In the univariate binary logistic regression analysis, hypertension, CVD, FBS, uACR, uNGAL, and uMCP-1 were found to be significantly associated with rapid progression. On multivariate regression analysis with all the significant univariate variables shown in Table 6, the uACR (odds ratio 1.0, 95% CI 0.99–1.0, *p* = 0.03), uNGAL (odds ratio 1.08, 95% CI 1.03–1.13, *p* = 0.01) and uMCP-1 (odds ratio 1.01, 95% CI 1.01–1.02, *p* = 0.01) were found to be independent risk factors for rapid progression. Post hoc power analysis for multiple regression analysis for NGAL and MCP-1 revealed powers of 0.08 and 0.086, respectively.

**Table 6.** Multivariate logistic regression analysis of rapid progression.

Variables	Univariate OR (95% CI)	*P* Value	Multivariate OR (95% CI)	*P* Value
Hypertension	0.128 (0.29–0.571)	***0.01***	0.549 (.039–1.69)	0.65
CVD	0.363 (0.18–0.72)	***0.02***	0.281 (.06–1.0)	0.06
SBP	.697 (0.45–1.05)	0.47	–	–
FBS	0.99 (0.45–1.2)	***0.01***	1.0 (0.99–1.0)	0.55
uACR	1.0 (0.9–1.0)	***0.01***	1.0 (0.99–1.0)	***0.03***
uNGAL	1.08 (1.05–1.1)	***0.01***	1.08 (1.03–1.13)	***0.01***
uMCP-1	1.01 (1.01–1.02)	***0.01***	1.01 (1.01–1.02)	***0.01***

CVD, cardiovascular disease; SBP, systolic blood pressure; FBS, fasting blood sugar; uACR, urine albumin–creatinine ratio; uNGAL, urine neutrophil gelatinase-associated lipocalin; MCP-1, monocyte chemoattractant protein-1.

### Diagnostic performance of uNGAL and uMCP-1 in predicting rapid progression

**Figure 4.**
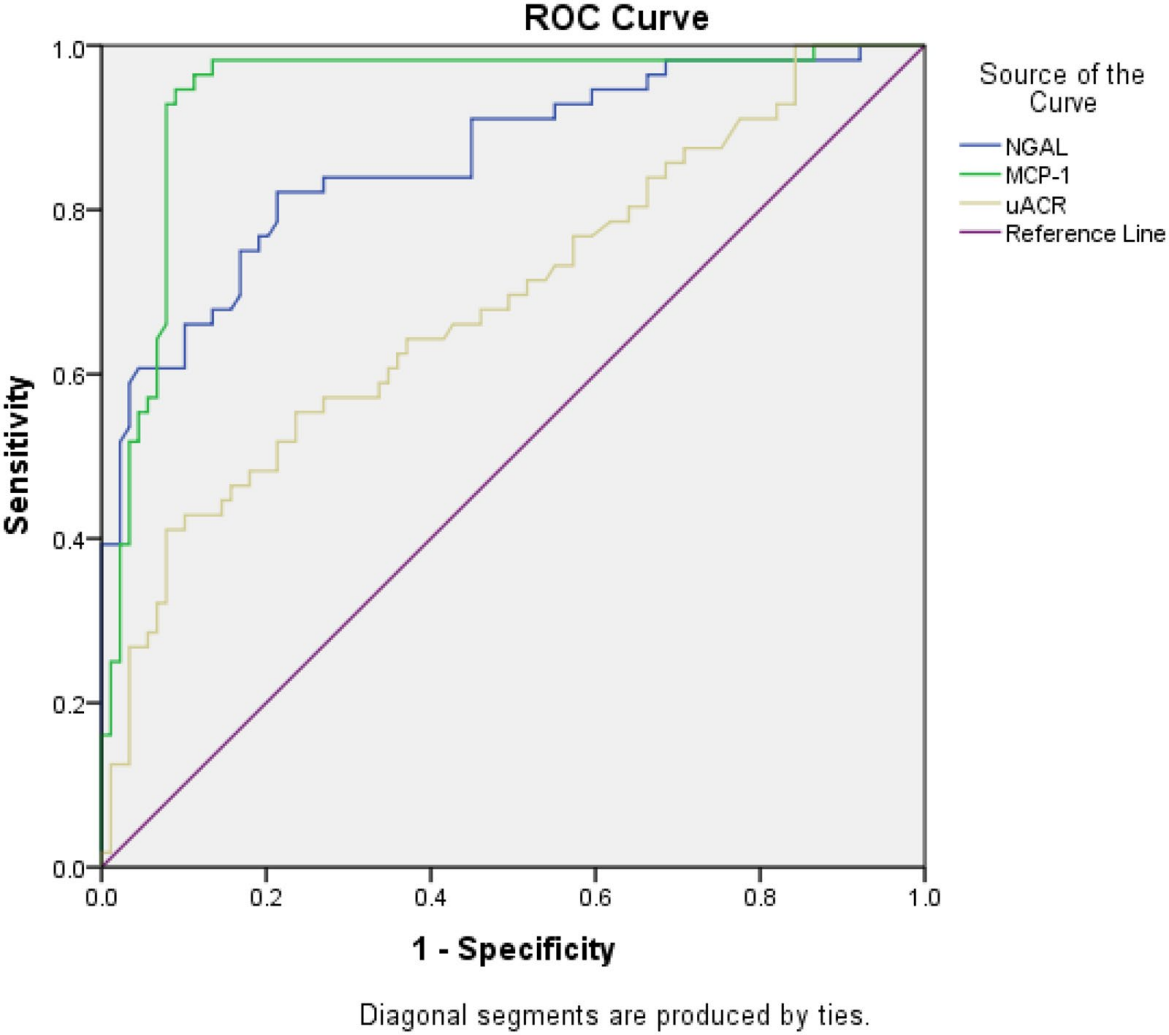
ROC curve showing the diagnostic performance of uNGAL, uMCP-1, and the uACR.

ROC analysis was performed (shown in Figure 4) to determine the diagnostic value of uNGAL and uMCP-1 in identifying DKD patients with or without rapid progression and the optimal cutoff values. uMCP-1 showed good diagnostic performance, with an AUC of 0.94 (95% CI: 0.88–0.98, *p* = 0.01), a sensitivity of 94.1%, and a specificity of 89.2%, with an optimal cutoff value of 381.2 pg/ml, and uNGAL showed an AUC of 0.86 (95% CI: 0.80–0.93), with 83.4% sensitivity and 77.3% specificity, with an optimal cutoff value of 39.8 ng/ml. The cutoff value of the uACR was 969 mg/g, with an AUC of 0.78 (95% CI: 0.699–0.872), 66% sensitivity and 98% specificity. We additionally assessed the combined predictability of uNGAL and uMCP-1 with the uACR, and the AUC of the three combined panels was better, 0.96 (Table 7).

**Table 7.** Combined AUC of biomarkers to predict rapid progression.

Biomarkers	AUC	*P* value
uMCP-1	0.94	0.01
uNGAL	0.86	0.01
uACR	0.78	0.01
uACR + uMCP-1	0.94	0.01
uACR + uNGAL	0.88	0.01
uACR + uNGAL + uMCP-1	0.96	0.01

## Discussion

Identifying DKD patients with rapid eGFR decline is essential to prevent disease progression to ESKD. The prevalence of rapid progression in our population was 38.6%, with a median eGFR decrease of −12 mL/min/1.73 m^2^/year. Similarly, a South Asian cross-sectional study reported that 30% of the study population experienced rapid progression to ESKD, with a mean eGFR loss of 17.5 ± 19.3 mL/min/1.73 m^2^/year [15]. Using a cutoff value similar to that used in our study, 28% were identified as rapid progressors [16]. Recently, 50.26% of rapid decliners were observed among Chinese DKD patients, with a median GFR slope of 8 mL/min/1.73 m^2^/year [2]. Furthermore, in a 10-year prospective study, 15.6% of DKD patients experienced a rapid decline [4]. The heterogeneity in the prevalence rate reported across the studies can be attributed to the variation in terms of defining rapid decline, some using absolute loss, and others in terms of percentage. Additionally, the multifactorial pathogenesis and diverse ethnic study population also contribute to this heterogeneity.

In our study cohorts, we observed a higher incidence of rapid progression among patients with baseline hypertension and CVD. This aligns with findings from recent studies indicating that patients with hypertension and CVD are more susceptible to rapid progression [4,17]. Zoppini et al. in a 10-year follow-up of 1682 patients with baseline eGFRs ≥60 mL/min per 1.73 m^2^/year reported that 93.9% of patients with hypertension had ≥4.0% annual GFR decline [4]. Likewise, a significant risk of disease progression was found in the hypertensive group in the RENAAL study [17]. Among the BP values, those with high systolic blood pressure experienced a rapid decline, which was consistent with previous findings that poor BP control is associated with DKD progression [17,18]. Chen et al. [19] reported a greater incidence of cardiac events over a 3-year follow-up period in eGFR decliners. Additionally, in line with the findings of Michael et al. our findings also revealed that ischemic heart disease was the major cause of cardiac events [20].

An analysis of biochemical parameters revealed that only the fasting blood sugar level and UACR were significantly higher in rapid progressors than in nonprogressors, which was consistent with the findings of Makoto et al. who reported that patients with poor DM control and proteinuria had a rapid decline in renal function [21]. After further stratification by the degree of proteinuria, the rate of progression was significantly high in those who had microalbuminuria (39.2%) and macroalbuminuria (57.1%). A small population within the normal or mildly increased albuminuria group (7.1%) also exhibited rapid progression, which supports the data from the UKPDS[22] and DEMAND [22], revealing the existence of renal function decline in those who do not exhibit proteinuria.

In the recommended medication regimen, those who were on the RAAS or SGLT2i were less likely to experience rapid progression. However, in our cohort, there were fewer patients on these drugs in both groups, and this difference was not statistically significant.

Tubular biomarkers constitute a sensitive index for assessing tubular damage. In this study, we measured the tubulointerstitial injury marker uNGAL and found a significant difference in the median uNGAL levels between rapid progressors and nonprogressors. This 25-kDa protein, which is part of the lipocalin superfamily, is abundantly released into the blood and urine by tubular cells, reflecting renal damage during DKD progression. Under physiological conditions, circulating NGAL is filtered by the glomeruli and almost entirely reabsorbed by proximal tubular epithelial cells. Elevated NGAL levels indicate ongoing renal damage and are associated with the progression of DKD [8]. Our finding is corroborated by studies from Giuseppe et al. and Bancha et al. who reported an increase in median uNGAL in the progressor group [23]. In contrast, Chou et al. reported that the mean uNGAL did not significantly differ among rapid progressors (18.3 ± 58.2 vs 23.3 ± 21.0) [24]. Further intergroup comparisons between albuminuria stages revealed a significant difference, with the highest median at macroalbuminuria and the lowest at normal albuminuria, which is in accordance with the findings of Kaul et al. who studied 144 type 2 diabetes patients with DKD among the Indian population and reported a similar trend of a progressive increase in uNGAL with worsening proteinuria [25]. This finding was further supported by Bolgano et al. who reported a consistent increase in uNGAL across the stages of proteinuria [26]. However, a contradictory finding was reported by Kim et al. where DKD patients presented no significant difference between the A1 group and the A2 albuminuria group [27]. In risk factor analysis with other clinical factors, the uNGAL level remained a strong independent risk factor for rapid progression, which is consistent with the findings of Kaul et al. [25]. ROC analysis revealed that uNGAL was more sensitive than specific, with a cutoff value of 39.8 ng/ml. Consistent with this, another prospective study involving 252 patients with DKD, which aimed at predicting rapid decline (>5 mL decline/year), similarly reported that uNGAL was less specific (48%) at a cutoff value of 614.4 ng/g [23]. However, Coppolino et al. reported good diagnostic performance for uNGAL, with the best cutoff value of 107 ng/ml and an AUC of 0.76 [28]. These findings highlight the strong association of the baseline tubular injury marker NGAL with the progression of DKD. NGAL is secreted by injured kidney cells and plays a regulatory role in tubular cell proliferation and apoptosis. It mediates signaling through the epidermal growth factor receptor, which in turn activates hypoxia-inducible factor 1-alpha, ultimately promoting cell proliferation, cytogenesis, renal injury, and the progression of DKD, suggesting that NGAL may represent a surrogate index of renal function decline in DKD [9].

MCP-1 is a potent chemokine that plays a crucial role in recruiting monocytes to the tubulointerstitium in DKD. The infiltration of inflammatory cells into renal tissue is a key contributor to the progression of DKD, as it promotes a proinflammatory microenvironment that intensifies tissue damage and drives fibrosis. In DKD, MCP-1 protein and mRNA expression have been detected in cortical tubules and infiltrating mononuclear cells. Moreover, urinary MCP-1 levels are positively correlated with the severity of both tubulointerstitial and glomerular lesions [6,7]. Consistent with these findings, our study also revealed significantly higher median urinary MCP-1 levels in rapid progressors (469 pg/ml) than in nonprogressors (220 pg/ml), corroborating the results reported by Natalia et al. [29], who reported that elevated uMCP-1 was associated with *a* ≥ 30% annual decline in the eGFR, with a median uMCP-1 of 542 pg/ml. This association was further supported by Satirapoj et al. who reported that uMCP-1 levels were increased in patients who had *a* ≥ 25% yearly decline in the eGFR [23]. These clinical observations align with the proposed pathophysiological mechanism whereby the diabetic milieu induces MCP-1 production by activating the NF-κB pathway, which promotes macrophage recruitment to the renal interstitium. This initiates inflammatory responses that contribute to the progression of DKD, leading to a decline in renal function [30]. In addition, we observed a significant gradual increase in uMCP-1 in the albuminuria group A1, A2 and to A3 in the intergroup comparison, which corroborates the findings of Bassayani et al. [31]. This elevated urinary excretion of MCP-1 in overt proteinuria is likely attributed to its upregulated production in renal tubules, possibly triggered by plasma proteins filtered by damaged glomeruli [30]. In contrast, Wada et al. reported a nonsignificant difference in the level of MCP-1 with worsening proteinuria, suggesting that proteinuria does not induce a significant increase in the level of MCP-1 [32]. Multivariate analysis revealed that, along with the uACR, the uMCP-1 concentration was an independent risk factor for rapid progression, which is consistent with the findings of Satirapoj et al. [23]. Several clinical studies have provided substantial evidence confirming the significant predictive role of uMCP-1, along with the uACR, in the decline in renal function in DKD patients [33]. Furthermore, ROC analysis indicated that uMCP-1 exhibited better diagnostic accuracy than did uNGAL or the uACR, with a sensitivity of 94.1%, a specificity of 89.2%, and an optimal cutoff value of 381.2 pg/ml. In the Canadian population, among a panel of other inflammatory markers, only uMCP-1 exhibited better predictability of renal function decline among patients with overt nephropathy, corroborating our results [29]. The cutoff value for predicting *a* ≥ 25% annual decrease in the eGFR was reported as 2.1 ng/mgCr, with an AUC of 0.73. Consistent with these studies, the ACCORD trial assessed the association between uMCP-1 and sustained GFR decline and was found to be fivefold more likely than a tubular injury marker to predict >40% GFR decline over 5 years [34]. Notably, considering these findings together, uMCP-1 is a promising marker for predicting rapid progression and confirming the close correlation of MCP-1-mediated inflammatory injuries involved in disease progression, which is of clinical value for the prognostic assessment of DKD. Furthermore, in the present study, the addition of urinary MCP-1 and NGAL to the traditional marker uACR slightly improved the predictive performance (AUC 0.96) compared with the use of uMCP-1 alone (AUC 0.94). Notably, this combined panel demonstrated substantially better diagnostic performance than the uACR alone (AUC 0.78). This finding corroborates the findings of Wu et al. who used a model that combines MCP-1 and the uACR with a tubular marker [35]. However, the diagnostic performance of better studies exploring the combined predictability of uMCP-1, uNGAL, and the uACR is limited, which warrants further validation.

Our study highlights the multifactorial pathogenesis of DKD and sheds light on the existing knowledge gap and lack of evidence on the ability of biomarkers from key pathways involved in DKD progression to predict rapid progression. To our knowledge, this is the first study to evaluate the role of NGAL and MCP-1 both individually and as a combined panel alongside the uACR. In this study, although both biomarkers were independent risk factors for rapid DKD progression, uMCP-1 showed better diagnostic performance than NGAL in terms of prediction, suggesting that uMCP-1 may be a promising biomarker for predicting rapid eGFR decline in DKD patients. However, further studies are needed to validate its added value over conventional markers such as the uACR. These results have important clinical implications, as uMCP-1 could aid in identifying high-risk patients for enrollment in clinical trials or for targeted, intensified interventions. These findings also provide further evidence supporting the role of MCP-1 in the pathogenesis of human DKD progression. This is particularly significant given that specific MCP-1 inhibitors are now available for clinical studies, offering the potential to reduce proteinuria [23]. Furthermore, in our cohort of patients with normal or mildly increased albuminuria, those who exhibited rapid progression had significantly higher uNGAL and uMCP-1 levels. In DKD patients with proteinuria, increased interstitial fibrosis correlates with declining renal function, and this decline occurs independently of albuminuria, suggesting that interstitial injury contributes in part to eGFR reduction [36]. In patients with normal albuminuria renal insufficiency, interstitial or vascular changes are more common than are the classic glomerular lesions of DKD, suggesting that tubulointerstitial injury may play a key role in its pathogenesis. NGAL is a low-molecular-weight biomarker of tubular injury that is freely filtered by the glomeruli and normally reabsorbed by the proximal tubules. In patients with T2DM and normal or mildly increased albuminuria renal insufficiency, tubular injury impairs reabsorption, leading to increased urinary excretion. We also suppose that the increased chemokine gradient formed in the tubulointerstitium promotes sustained monocyte infiltration into the kidney, driving a persistent inflammatory response until the development of clinical-onset DKD [37]. This highlights the clear need to explore novel biomarkers that can more reliably identify individuals at risk for adverse renal outcomes. MCP-1 and NGAL are logical candidate biomarkers, as they play a central role in the pathogenesis of renal fibrosis and are readily measurable in urine [38]. On the basis of these findings, our study evaluated the combined panel analysis of tubular injury markers and inflammatory markers in addition to the conventional biomarker albuminuria (0.96) and revealed better predictability than did the use of the uACR alone (AUC 0.78), which could improve the accuracy in predicting rapid progression. However, larger studies are needed to assess the cost-effectiveness of the addition of uMCP-1 and NGAL measurements for the prediction of adverse renal outcomes in this population [39].

The limitations of this study include its single-center observational design and the absence of a comparison of NGAL and MCP-1 levels with those of healthy controls. Additionally, the study did not include estimations at two time points (baseline and follow-up). Data on renal endpoints such as the need for renal replacement therapy and mortality could not be obtained because of the short follow-up period.

## Conclusion

A significant proportion of type 2 DKD patients in our population had rapid progression. The presence of hypertension, cardiovascular disease, high fasting blood sugar, and high SBP were significantly associated with this acceleration. While uACR remains a conventional biomarker, its diagnostic accuracy is limited in certain clinical scenarios, underscoring the need to explore novel biomarkers for more precise prediction of DKD progression. Our findings demonstrate that uNGAL and uMCP-1 levels are elevated in rapid progressors and increase progressively with worsening albuminuria. Both biomarkers, along with uACR, emerged as independent predictors of rapid decline, with uMCP-1 showing superior diagnostic performance characterized by higher sensitivity and specificity. The combined assessment of uMCP-1, uNGAL, and uACR significantly enhanced predictive accuracy, emphasizing the importance of multipanel biomarker strategies for better risk stratification. Incorporating these biomarkers into routine clinical practice could facilitate earlier detection of high-risk patients, enabling more timely and targeted therapeutic interventions to potentially mitigate disease. However, larger, multi-center studies with longer follow-up are needed to validate these findings.

## Data Availability

All the data generated or analyzed during this study are included in this article. Further inquiries can be directed to the corresponding author.
